# Membrane Affinity of Platensimycin and Its Dialkylamine Analogs

**DOI:** 10.3390/ijms160817909

**Published:** 2015-08-04

**Authors:** Ian Rowe, Min Guo, Anthony Yasmann, Abigail Cember, Herman O. Sintim, Sergei Sukharev

**Affiliations:** 1 Department of Biology, University of Maryland, College Park, MD 20742, USA; E-Mails: ianrowe@umd.edu (I.R.); ayasmann@gmail.com (A.Y.); 2 Department of Chemistry and Biochemistry, University of Maryland, College Park, MD 20742, USA; E-Mails: mguo@umd.edu (M.G.); hsintim@umd.edu (H.O.S.); 3 Biochemistry and Molecular Biophysics Graduate Group, University of Pennsylvania, Philadelphia, PA 19104, USA; E-Mail: cember@mail.med.upenn.edu; 4 Maryland Biophysics Program, University of Maryland, College Park, MD 20742, USA

**Keywords:** membrane permeability, drug insertion, hydrophobicity, amphipathicity, monolayers, lateral pressure, mechanosensitive channel

## Abstract

Membrane permeability is a desired property in drug design, but there have been difficulties in quantifying the direct drug partitioning into native membranes. Platensimycin (PL) is a new promising antibiotic whose biosynthetic production is costly. Six dialkylamine analogs of PL were synthesized with identical pharmacophores but different side chains; five of them were found inactive. To address the possibility that their activity is limited by the permeation step, we calculated polarity, measured surface activity and the ability to insert into the phospholipid monolayers. The partitioning of PL and the analogs into the cytoplasmic membrane of *E. coli* was assessed by activation curve shifts of a re-engineered mechanosensitive channel, MscS, in patch-clamp experiments. Despite predicted differences in polarity, the affinities to lipid monolayers and native membranes were comparable for most of the analogs. For PL and the di-myrtenyl analog QD-11, both carrying bulky sidechains, the affinity for the native membrane was lower than for monolayers (half-membranes), signifying that intercalation must overcome the lateral pressure of the bilayer. We conclude that the biological activity among the studied PL analogs is unlikely to be limited by their membrane permeability. We also discuss the capacity of endogenous tension-activated channels to detect asymmetric partitioning of exogenous substances into the native bacterial membrane and the different contributions to the thermodynamic force which drives permeation.

## 1. Introduction

Permeation through the membrane is the first step in the mechanism of any drug that targets intracellular components or processes. Balanced hydrophobicity of the drug, while keeping sufficient solubility, usually confers direct permeability through the lipid bilayer without requiring a special transport mechanism [1]. Generally hydrophobicity, measured as an oil-water or octanol-water partitioning coefficient (logP), reasonably correlates with membrane solubility and permeability [2]. While the equilibrium measurements of two-phase partitioning take a long time, reverse phase HPLC on hydrophobic media [3] may give a faster answer. Uneven distribution of polar and apolar groups define amphipathicity of a substance, *i.e.*, propensity toward an interface, and is quantified as the surface activity at the air-water interface [4]. Amphipathicity was also found to be a good corollary of the general membrane and blood-brain barrier permeability [5]. Neither an oil-water system nor an air-water interface, however, accurately represent the membrane or account for drug-phospholipid interactions. A system based on phospholipid-impregnated filters (PAMPA) [6] mimics membrane composition well but may not precisely recreate the bilayer structure. The lipid (Langmuir) monolayers previously used to assess affinities of anesthetics [7,8] represent only half a membrane with artificially controlled lateral pressure and lipid density, which are variables effecting drug partitioning. Direct partitioning into native cellular membranes can be measured in a straightforward fashion with radioactive, spin-labeled, or fluorescent compounds. However, partitioning cannot be easily assessed for any arbitrary substance of interest.

**Figure 1:**
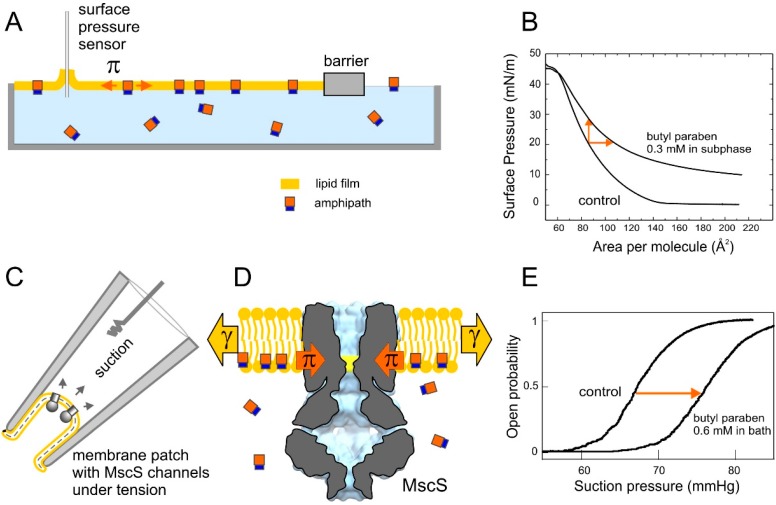
Experimental systems used for studies of lateral pressure perturbations by amphipathic drugs; (**A**) Langmuir monolayer with amphipathic substance in the subphase. (**B**) Two compression isotherms obtained with *E. coli* polar lipids as the control and in the presence of the amphipath butyl paraben. The upward shift of isotherm signifies additional pressure in the film due to the intercalation of paraben; (**C**) Excised patch configuration for electrical recording of MscS population currents evoked by pressure ramps. Applied negative pressure to the pipette (suction) produces tension in the patch membrane; (**D**) Cartoon depicting the intercalation of amphipaths into the inner leaflet of the bilayer and exerting a lateral pressure on MscS; and (**E**) The intercalating amphipath exerts additional lateral pressure in the inner leaflet of the patch membrane and shifts the activation curve to the right.

Early patch-clamp recordings in giant bacterial spheroplasts indicated that amphipathic substances known to deform erythrocytes (crenators), such as chlorpromazine or trinitrophenol, also change the open probability of bacterial mechanosensitive channels. The change in open probability is due to unilateral intercalation into the lipid bilayer [9,10]. This observation was initially interpreted as a curvature-inducing effect of amphipaths. Later analysis suggested that, independent of the spontaneous curvature the substances induce, insertion of new material into one leaflet will lead to a redistribution of tension between the leaflets. This redistribution may explain the observed changes in channel activity [11]. When the mechanosensitive channel of small conductance from *E. coli*, MscS, was first crystallized [12], the cytoplasmic position of the gate relative to the mid-plane of the membrane suggested a greater sensitivity to tension/lateral pressure in the inner leaflet as opposed to the outer. This notion was supported by experiments involving an asymmetric addition of trifluoroethanol [13], which shifted activation curves to the right when presented to the cytoplasmic side and to the left when incorporated into the extracellular leaflet. A comparative study of three esters of parabenzoic acid (parabens) indicated that the magnitude of right shift of the MscS activation curve reflected the extra tension needed for channel opening. This extra tension closely correlated with the lateral pressure exerted by intercalation of these substances into the monolayer formed of bacterial lipids [14] (see Figure 1). The observation of activation curve shifts followed by a return to the original position was interpreted as a time-dependent redistribution of the intercalating substance to the opposite leaflet (*i.e.*, permeation), leading to a restoration of lateral pressure symmetry. A subsequent study of autoinducers (AI-1, AI-2, indole) in these two systems confirmed the ability of MscS to detect lateral pressure changes caused by the partitioning and permeation of amphipathic quorum signaling molecules across the inner bacterial membrane [15].

In the present study we performed a further comparison of this electrophysiology-based approach with traditional surface chemistry techniques. We optimized MscS as a sensor of lateral pressure asymmetry by introducing a mild gain-of-function mutation that increases its sensitivity. Then, we focused on the membrane partitioning of platensimycin (PL) and six synthetic analogs. This broad-spectrum antibiotic targets β-ketoacyl synthases (FabF/B) in prokaryotes, as well as eukaryotic fatty-acid producing enzymes [16,17,18]. The high cost of its biosynthetic production drives the development of synthetic analogs. For the six dialkylamine analogs of PL which were synthesized with similar dihydroxyl benzoate pharmacophores but different side chains, biological activity varied by more than two orders of magnitude [19].

To understand this difference in activity between the analogs, we assessed their binding affinities to FabF using computational docking. In parallel, to estimate whether their antibacterial efficiency could be limited by low membrane permeability, we performed computations of their polarity/hydrophobicity and octanol-water partitioning coefficients with several algorithms. We then assessed membrane partitioning for PL and its analogs experimentally by measuring surface activity and the ability to intercalate into phospholipid monolayers and native bacterial membranes for each compound. Patch-clamp measurements on *E. coli* membranes were facilitated by a re-designed MscS used as a lateral pressure sensor. Despite highly varied computational predictions, all experimental techniques gave comparable partitioning parameters for the analogs. Among the experimental techniques, we observed modest deviations of partitioning parameters for two compounds with bulky sidechains (the di-myrtenyl QD-11 and platensimycin itself). The comparison of data obtained with the three techniques suggests that although the common hydrophobic effect might be the main contribution driving the substances from water to the membrane interface, the effect of favorable interactions with phospholipids cannot be ignored for these largely aromatic compounds. The role of lateral pressure of lipids in reducing the affinity is seen in the comparison of monolayer experiments with patch-clamp trials characterizing intercalation into native bilayers. The study permits a more informed approach in optimizing drug lipophilicity/amphipathicity for best delivery into the cell.

## 2. Results

### 2.1. Optimizing MscS as a Lateral Pressure Sensing Device

MscS was previously used as a lateral pressure sensor to study paraben partitioning into the inner *E. coli* membrane [14]. These studies revealed direct correlations between the shifts of pressure midpoints (p_0.5_) for MscS activation and surface pressure created by substances due to intercalation into lipid monolayers. This suggested that the pressure sensor of the Langmuir apparatus and MscS detect the same parameter of lateral pressure change. In contrast to the Langmuir monolayer system, MscS senses not just the absolute value of lateral pressure, but also the difference in pressures between the two membrane leaflets (Figure 1) because the channel spans across both leaflets but has the gate positioned at the interface of the cytoplasmic monolayer. The drawback of the original patch-clamp system employing WT MscS expressed in the MJF465 *E. coli* strain was that positive shifts of activation curves required application of higher tensions, which destabilized the patches. Another problem was the massive amount of MscS inactivation upon exposure to the concentrations of intercalating agents required to produce large shifts [15].

**Figure 2:**
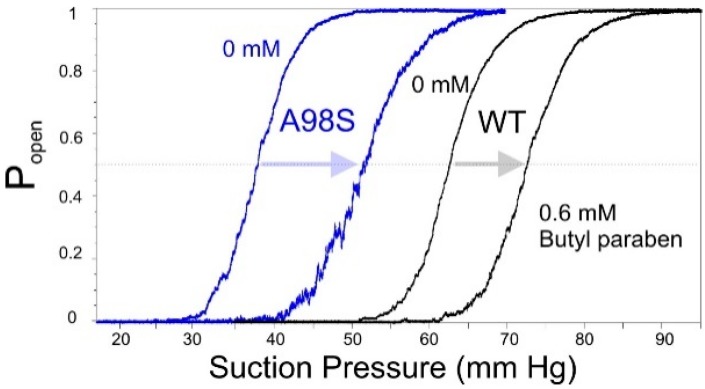
The mild gain-of-function A98S mutation of MscS increases its sensitivity to asymmetrically intercalating agents. A patch-clamp experiment showing the activation curves in response to a 1 s ramp of pressure for both WT (black traces) and A98S (purple). Upon exposure to 0.6 mM butyl paraben, the activation curve of both patches shifts to the right, with the greatest change seen for A98S.

We explored the A98S gain-of-function mutant of MscS and found it to be a more suitable sensor. In pressure ramp experiments this mutant activates earlier, but apparently has the same tension dependency of inactivation as WT [20]. The two competing processes of opening and inactivation both begin in the resting state (open channels do not inactivate). When channels have lower tension thresholds for activation, opening becomes a preferred path and these mutants generally do not inactivate [21]. The serine-for-alanine substitution hydrophilizes the outer portion of the hydrophobic pore. As a result, the remaining hydrophobic region is localized to the cytoplasmic portion of the pore, placing the gate more asymmetrically relative to the mid-plane of the bilayer. This mutant activates at 5.8 mN/m (p_0.5_) *vs.* 7.8 mN/m for WT MscS [21], and exhibits reduced inactivation. We tested this mutant against WT with a cytoplasmic addition of 0.6 mM paraben, which has been characterized previously [14]. The typical activation curves (open probability *vs.* pipette pressure) measured with pipettes of equal size for the two channels are shown in Figure 2. Not only is the absolute shift of activating pressure (p_0.5_) greater for A98S, but the percentage of midpoint shift relative to the original midpoint averaged for six independent patches was 41% ± 6% for the mutant *vs.* 14% ± 4% for WT. This illustrates the higher sensitivity of the mutant to a given lateral pressure perturbation.

### 2.2. Synthetic Analogs of Platensimycin, Their Biological Activity and Predicted Affinities to FabF

**Figure 3:**
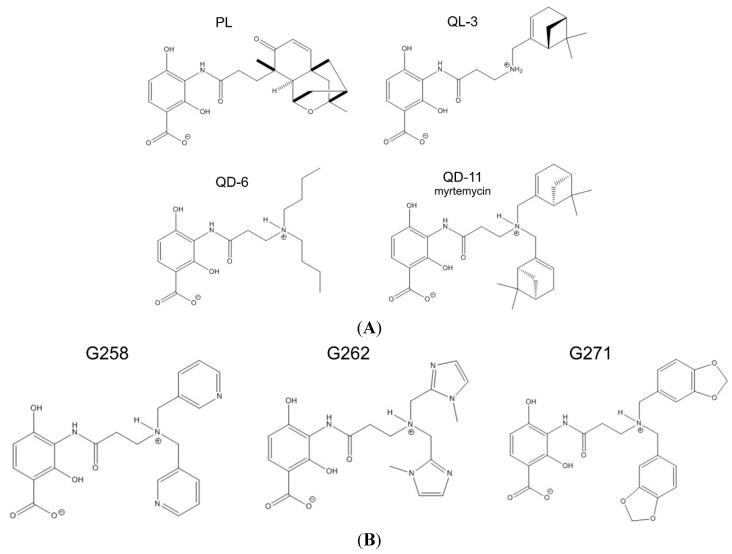
The chemical structures of the first generation of dialkylamine analogs of platensimycin (PL). (**A**) See more details in supplemental Figure S1A; and (**B**) See more details regarding charge distribution in supplementary Figure S1B.

**Scheme 1:**
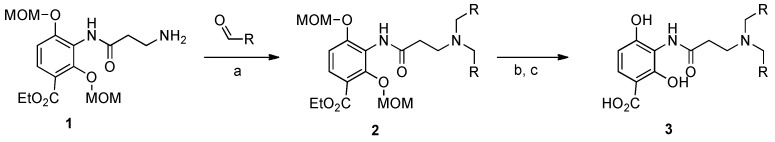
Synthesis of platensimycin analogs. (**1**) Aldehyde (3 equiv.), NaCNBH_3_ (1.5 equiv.), HOAc (3 equiv.), EtOH, RT, 1.5 h; (**2**) NaOH (4 equiv.), EtOH/H_2_O (*v*/*v*) = 3/1, 40 °C, 7 h; (**3**) 6 M HCl aq. (20 equiv.), 40 °C, 6 h.

Having identified the A98S MscS mutant as a more sensitive sensor than WT, we proceeded to investigate the permeation of platensimycin and analogs across the bacterial membrane. All of the platensimycin analogs described herein share the same benzoic acid pharmacophore and ethylene linker, but vary at the side chain. Figure 3 presents the structures of platensimycin and the six analogs. The amino group in compound 1 (Scheme 1, see Experimental Section) facilitated the installation of alkyl and aryl groups of different hydrophobicity via reductive amination. The easy installation of alkyl groups on compound 1 contrasts with the difficult synthesis of the tetracyclic core in platensimycin, which involved more than 10 synthetic steps and multiple chromatographic separations. QD-06, QL-03, and QD-11 (myrtemycin) (Figure 3A) were the first generation of platensimycin analogs previously reported [19].

Platensimycin has a bulky non-planar tetracyclic sidechain with two oxygens, which confer polarity. QD-06 has two simple linear butyl chains, QL-03 contains one myrtenyl ring, and QD-11 (myrtemycin) contains two bulky myrtenyl rings. The second generation of analogs (Figure 3B) was synthesized with planar heterocyclic sidechains which were supposed to provide aromatic surfaces for possible interactions and provide balance between the hydrophobicity and solubility.

Principally, the efficacy of a drug depends on permeability (first selection), the degree of inhibition of the biological target, rates of drug efflux, drug breakdown, off target binding, and sequestration. In the case of platensimycin, the biological target is FabF. Computational docking experiments (Figure S2 and Table 1) suggested that three analogs (QD-11, G258, and G271) may potentially have similar affinities for FabF as platensimycin. Details of docked conformations are presented in the Supplementary Figure S2A,B. Platensimycin and QD-11 show the same estimated binding affinity to FabF (C163Q) (PDB ID: 2GFX) and both are potent antibiotics. QL-03 and QD-06 could not be successfully docked into the enzyme’s binding pocket and for this reason, the data are not presented in the table. G258 and G262 exhibited weaker binding affinity to FabF, and consistent with computations, they had essentially no biological activity. For G271 a similar binding affinity was predicted as for platensimycin and QD-11, however in experiments this analog showed no antibiotic activity. More specifically, platensimycin and myrtemycin were active against Gram-positive bacteria (*S. aureus*, MRSA; *E. faecium*, VRE and *B. subtilis*) and also Gram-negative bacteria, *E. coli* (but only when co-adminsitered with Phe-Arg-β-naphthylamide dihydrochloride, PAβND, which is a bacterial efflux pump inhibitor) as described in [18]. For the purpose of comparison with the computational docking predictions, here we present data for *B. subtilis*.

**Table 1:** Calculated binding affinities of PL analogs for FabF (C163Q) (PDB ID: 2GFX) and experimental minimal inhibitory concentration (MIC)s of these compounds determined with cultures of *Bacillus subtilis* [22].

Drug Activity Parameter	PL	QL-03	QD-06	QD-11	G258	G262	G271
Computed affinity (kcal/mol)	9.1	n/a	n/a	9.1	8.0	7.7	8.9
MIC for *B. subtilis* (µg/mL)	2	>256	128	4	>512	>512	>512

Because only QD-11 was found active against bacteria, we hypothesized that the inactivity of G258 and G271 could be caused by low membrane permeability, whereas the low activity of QL-3, QD-6, and G262 could be caused by the combined effect of low affinity for FabF and possibly low membrane permeability. Before embarking on experimental measurements of membrane partitioning, we estimated polarity for all of these compounds computationally.

### 2.3. Computational Assessment of Polarity and Membrane Partitioning

To develop an initial estimate on membrane partitioning, we calculated the pKa of ionizable groups using the *Chemicalize* server and thus prepared the system for quantum calculations of partial charges. At physiological pH the carboxyl group on the dihydrobezoic acid, common for platencimysin and the analogs, is predicted to be in the deprotonated state (pKa~3). However, in contrast to platencimysin, all analogs have a tertiary amine that is predicted to be fully protonated under similar conditions (pKa ~10). Therefore the zwitterionic characteristic of the analogs may potentially influence their membrane-water distribution.

Gaussian calculations (Supplementary Figure S1A,B) predicted partial charge distribution and the percentage of polar solvent-accessible area for each molecule (Table 2, Column 2). For platensimycin, the polar area constituted 36% of the molecule. Partial charges were largely distributed around the benzoic acid and partially on the oxygen atom of the tetracyclic ring. In QD-06, QD-11, and QL-03 partial charges are mainly on the benzoic acid as well as on the protonated tertiary amine nitrogen. QD-06 and QD-11 are both more hydrophobic than platensimycin, with the percentage polar areas of 30% and 25% respectively. The calculated hydrophobicity of QL-03 (percentage polar area of 34%) is similar to platensimycin. For G258, G262, and G271 the side chains contain heteroatoms and hence partial charges are distributed on both the benzoic acid, nitrogen, and the side chain. Among these three, G271 turned out to be most hydrophobic.

**Figure 4:**
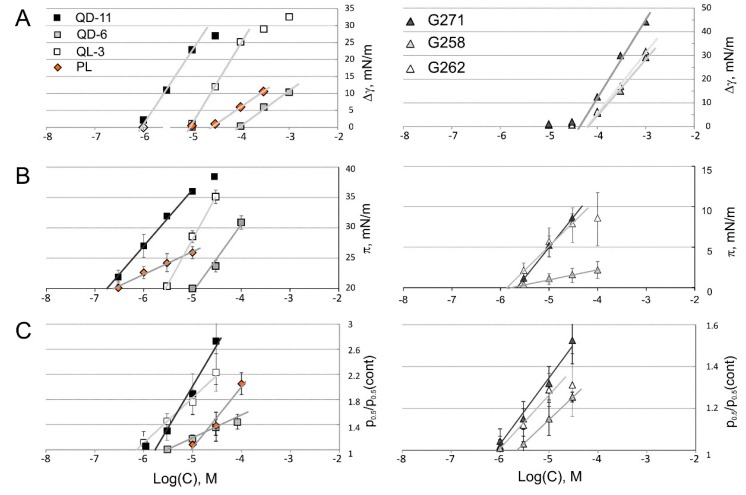
Shifts of the MscS activation curve correlate to changes in surface tension and lateral pressure in Langmuir monolayers. The changes in surface tension (∆γ, panels **A**), lateral pressure (∆π, panels **B**), and activation midpoint (p_0.5_) of MscS normalized to p_0.5_ in control (**C**) are plotted against the logarithm of drug concentration. The plots on the left represent those obtained from platensimycin (PL), QD-6, QL-3 and QD-11. The plots on the right represent heterocyclic compounds. The straight lines represent fits of linear parts of the curves where x-intercept corresponds to the inverse partitioning coefficient in each system (see Experimental Section). In some instances fitting was done using only three points due to non-linearity of the curve at the foot, or due to micellation at higher concentration. The *R*^2^ parameter reflecting quality of fitting in all cases was higher than 0.98 in all trials.

In parallel, we utilized two web-based algorithms predicting octanol-water partitioning coefficients logP. Here we call this parameter log*K*_OW_ to specify the two phases. *ALOGPS* [23] and *Molinspiration* [24] programs “trained” on compound libraries, predict log*K*_OW_ based on the type, number, and positions of polar atoms but taking no account of ionic state, whereas *Chemicalize* [25] provides values of logD that predict octanol-water partitioning with the account of all ionized groups. Log*K*_OW_ gave the ranking of substances relative to moderately hydrophobic platensimycin, with QD-11 being the most hydrophobic and G262 the least hydrophobic. LogD gave essentially the same ranking but the absolute values were 2–3 orders of magnitude lower. As will be shown below, experimental log*K*_mem_ values measured at pH 7.2 are closer to log*K*_OW,_ suggesting that membrane partitioning of these substances is determined by factors other than the ionization state in aqueous solution. The experiments presented below also show that all computational algorithms underestimate membrane partitioning. The comparison of all the data collected using tensiometry at the air-water interface, Langmuir technique, and patch-clamp measurements with A98S MscS is summarized in Figure 4. The resulting estimations of air-water (*K*_AW_), lipid monolayer (*K*_lip_), and membrane (*K*_mem_) partitioning coefficients for platensimycin (PL) and the six synthetic analogs are presented in Table 2 along with the predicted log*K*_OW_ and logD. Below we provide some details and illustrations on how this data was obtained.

**Table 2:** Calculated and experimentally estimated partitioning parameters for platensimycin and its three analogs, compared with the anti-microbial activity. Columns 3 and 4 are computational predictions (designated by *) by two programs of the oil-water partitioning coefficient (*K*_OW_) based on the exposed polar area of the molecule. Column 5 shows computational estimation of logD parameter predicting the partitioning from water into octanol with the account of all ionized aqueous species present in the mixture. Column 6 is the air-water (*K*_AW_) partitioning coefficient obtained from tensiometry. The lipid partitioning coefficients (*K*_lip_) were estimated from monolayer pressure-area isotherms @ 20 mN/m (Column 7) and values corrected for 35 mN/m are in Column 8. Molecular area for each compound (Column 9) was estimated from the slopes of re-partitioning back to the subphase with surface pressure (see Experimental Section and Figure 5 and Figure 6). Log*K*_mem_ (Column 10) were obtained from the x-intercepts of midpoint shift (∆p_0.5_) *vs.* concentration curves obtained in patch-clamp experiments (Figure 4).

1	2	3	4	5	6	7	8	9	10
Compound	Polar/Total Area, Å^2^	ALOGPS log *K*_OW_ *	Molinspiration log *K*_OW_ *	Chemicalize logD	log *K*_AW_ (tens.)	log *K*_lip_ @ 20 mN/m, Measured	log *K*_lip_ @ 35 mN/m, Corrected	Effective Molec. Area, Å^2^	log *K*_mem_ (patch-clamp)
QD-11	110/440, 25%	7.44	6.07	4.09	6.1	6.7	6.2	31	5.7
G271	159/439, 36%	4.11	3.40	1.62	4.5	6.1	5.8	18	6.1
QD-06	110/366, 30%	3.52	3.70	1.81	4.1	5.0	4.5	30	5.5
QL-03	119/353, 34%	3.79	2.85	0.99	5.0	5.6	5.2	28	6.1
PL	133/373, 36%	3.32	2.84	0.02	4.6	6.6	6.1	31	5.0
G258	136/374, 36%	0.89	1.15	−0.18	4.2	5.9	5.6	18	5.6
G262	141/387, 36%	0.59	0.46	−0.18	4.2	5.9	5.6	17	5.6

### 2.4. Tensiometry

Tensiometry experiments for QD-6, for which a decrease of surface tension was observed only in the sub-millimolar range, produced a linear Δγ(logC) dependence and a log*K*_AW_ of about 4.0 (Figure 4A). For the less hydrophobic G258 and G262, changes of surface tension were also detected only at 10^−4^ M (Figure 4B). QL-3 and especially QD-11, containing one and two myrtenyl rings, were considerably more surface-active with a log*K*_AW_ of 5.0 and 6.1, respectively. At higher concentrations, their Gibbs isotherms visibly rolled off, signifying micelleation/aggregation in water. Platensimycin, which is predicted to be more hydrophilic than QD-11, G271, and QD-6 (Table 2), produced no visible effect on surface tension up to 30 µM. The tensiometry data generally agrees with computed hydrophobicities with the exception of QL-3, which appeared more hydrophobic/amphipathic than predicted.

### 2.5. Langmuir Monolayer Experiments

**Figure 5:**
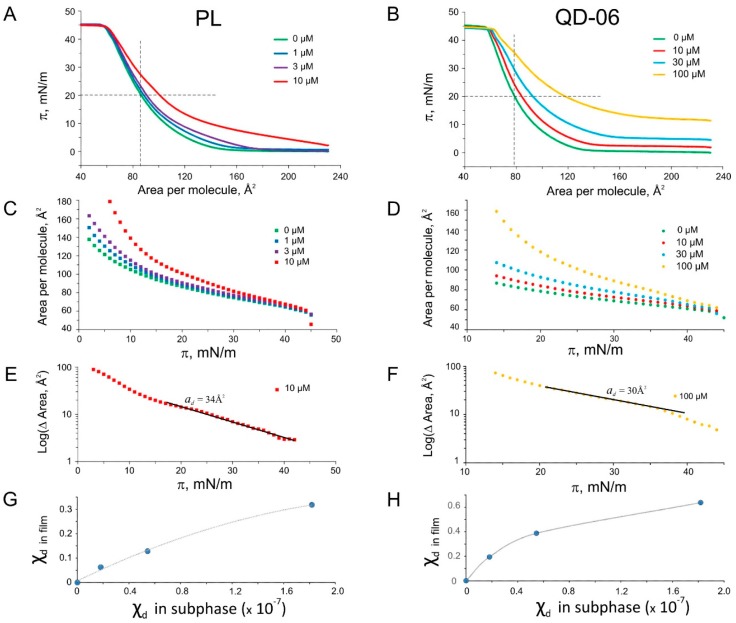
Pressure-area (π-A) isotherms for platensimycin and its aliphatic analog QD-6. Green curves in all cases are control isotherms taken without intercalating agents. The upward-right shifts of π-A curves with concentration (**A**,**B**); indicate intercalation of the amphipathic substance from the subphase into the monolayer. Plots of the same curves in inverted A-π coordinates (**C**,**D**); and differences from control presented in semi-log scale (**E**,**F**); with the linear fits and the estimated molecular areas for the drugs. Linear segments of the curves in panels E and F, covering the π ranges of between 20 and 40 mN/m were chosen for fitting. Mole fraction of drugs in the monolayers plotted *vs.* mole fraction in the subphase (**G**,**H**).

**Figure 6:**
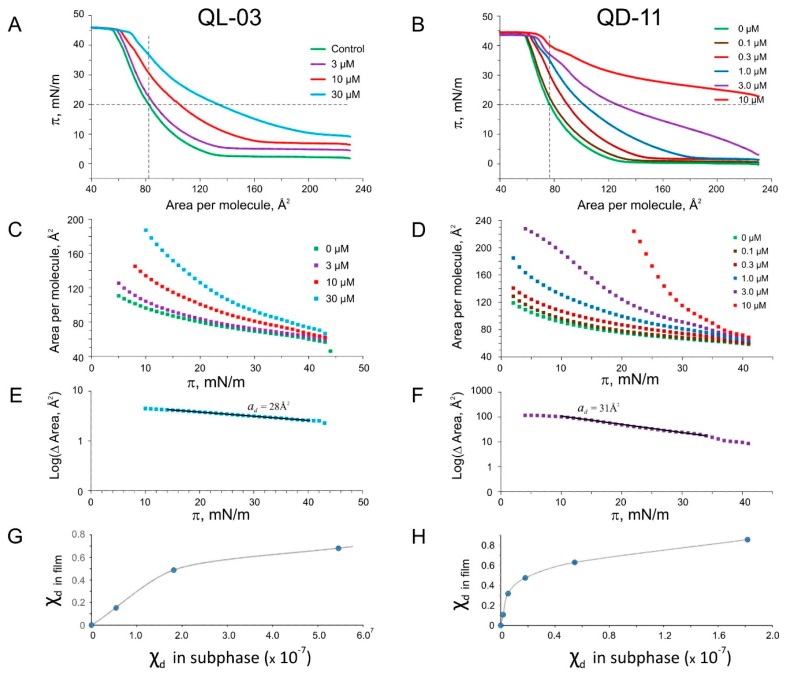
Pressure-area (π-A) isotherms for two myrtenyl platensimycin analogs, QL-3 and QD-11, and their analysis. The families of π-A isotherms taken at different drug concentrations in the subphase (**A**,**B**); which were then re-plotted in A-π coordinates (**C**,**D**); Molecular areas for the drugs are calculated from the slopes of semi-logarithmic plots of the area differences between experimental curves and the control (**E**,**F**); Mole fraction of the drugs in the monolayers plotted *vs.* the mole fraction in the subphase (**G**,**H**).

The families of isotherms for four compounds (platensimycin, QD-6, QL-3, and QD-11) are shown in Figure 5 and Figure 6 (panels A and B). The green lines represent control curves with no drug in the subphase. Upon increasing drug concentrations, the observed upward-right shifts of isotherms signify swelling of the film due to drug intercalation. One can see that it takes about one order of magnitude more of aliphatic QD-6 to produce comparable shifts of isotherm position compared to platensimycin. In these experiments we chose π = 20 mN/m as a reference point for measuring drug intercalation. At this pressure the control monolayer is less dense than a typical bilayer and the area per lipid is 86 Å^2^ instead of the regular bilayer packing area of 68 Å^2^, which is reached at the monolayer-bilayer equivalence pressure of 35 mN/m. By scoring pressures at this specific area while increasing platensimycin in the subphase, we created the plot shown in Figure 4B with orange diamonds. The x-intercept of the linear fit designates the inverse *K*_lip_ observed at that area per molecule. *K*_lip_ is highest for QD-11; platensimycin, despite the relatively low computational predictions of log*K*_OW_ (Table 2), comes second.

To further analyze this series of isotherms, we re-plotted them in area-pressure coordinates (Figure 5C,D) and calculated the area differences from the control as a function of pressure. The differences decrease with pressure, which reflects displacement of the drug from the monolayer back to the subphase. Plotted in log scale, these differences (log(ΔArea)) exhibit nearly linear segments which allowed us to fit the slopes and estimate molecular area (*a*_d_) taken by the drug in the monolayer. As explained here in the Methods section, the calculation of area works only at small mole fractions of the drug in the monolayer; however, at low drug concentrations the accuracy of Δ*A* measurements suffers. Generally, the slopes of log(Δ*A*) over π slightly increased with the drug concentration, and in Table 2 we present the values for the areas that were reliably measured at higher concentrations. The slopes consistently estimated *a*_d_ for all the analogs between 17 and 28 Å^2^, with the exception of bulky platensimycin and its double myrtenyl analog QD-11, covering 31–34 Å^2^ in the plane of the monolayer. We should remember that computed minimal cross-sectional areas for all these substances are larger (60–80 Å^2^). The discrepancy may come from the fact that these substances pack between lipids in a specific way, obeying drug-lipid interactions (like PC-cholesterol [26,27]) that minimizes their effective footprint. One should take into account that the degree of anisotropy and the lateral pressure profile in the monolayer are different from those in the bilayer [28] and for this reason the z-position of the drug may be different.

Analysis of individual isotherms taken at different drug concentration at π = 20 mN/m (horizontal cross-section of the family of curves) gave us measurements of the Δ*A*, reflecting the amount of new material partitioning into the film (monolayer dilation). With a simple assumption that Δ*A*/*A*0 *= a*_d_*·n*_d_/*a*_L_*·n*_L_ we estimated the mole fraction of each drug in the film and plotted it as a function of mole fraction in the bulk (Figure 5 and Figure 6G,H). With large monolayer dilation, the drug mole fraction in the film often exceeds 0.5 and the apparent partitioning coefficient *K*_lip_ decreases. The ${\text{χ}}_{\text{d}}^{\text{σ}}$(χ*_d_*) curves are vividly nonlinear. This suggests that drug-lipid interactions are considerably stronger than drug-drug interactions in the monolayer. As seen from Figure 6, QD-11, a compound with two bulky myrtenal rings, has a higher affinity for monolayer than its analog QL-3, with one myrtenal ring. The presence of QD-11 is detectable on isotherms at 10^−7^ M in the subphase. Similar analysis for the heterocyclic G258, G262, and G271 compounds is presented in the Supplementary Figure S3. All gave very similar log*K*_lip_~6.0, again despite computational predictions of oil-water partitioning coefficient based on the fraction of polar solvent-accessible area, log(*K*_OW_)~4 (Table 2). It is likely that in addition to hydrophobic exclusion from water, lipophilicity of these compounds is dominated by large areas of Van der Waals contact with lipids and neighboring drug molecules. At the highest studied concentrations, all substances remain in the film all the way to the collapse, which is 7–10 mN/m above the monolayer-bilayer equivalence pressure. This suggests that these substances have a substantial propensity toward the lipids and are able to wedge between the lipids in the native membrane that exists at the same packing density and lateral pressure. Similar shifts of monolayer isotherms due to partitioning and pressure-dependent equilibria have been previously reported for other substances [29,30,31].

### 2.6. Patch-Clamp Investigation of Drug Insertion into the Bacterial Cytoplasmic Membrane

**Figure 7:**
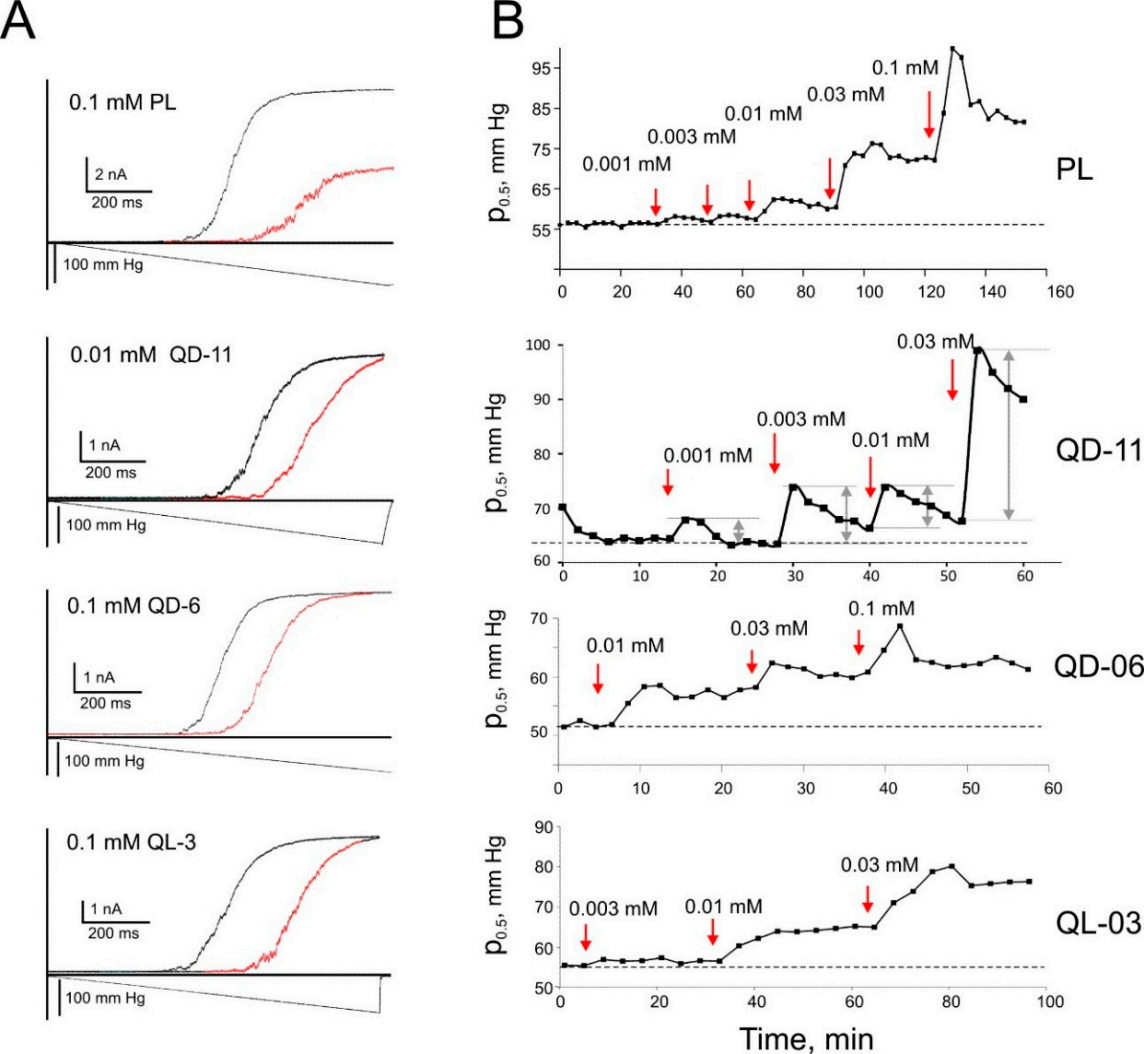
Ramp responses of A98S MscS following the addition of platensimycin and its analogs and the time dependencies of the resulting midpoint shift. The shift of the activation curve of A98S MscS toward higher tension after exposure to the denoted compounds is shown (**A**); The activation midpoints of the curves (p_0.5_) were then plotted against time (**B**). Red arrows indicate the point in time at which the given concentration of the compound was added to the bath; control injections of equivalent volumes of ethanol did not change the activation curve. Cumulative amplitudes of p_0.5_ shifts (shown by grey arrows in the plot for QD-11) were used to plot and fit concentration dependencies in Figure 4C.

The responses of isolated patches containing multiple A98S MscS channels to linear pressure ramps produced sigmoidal activation curves with saturation. The saturating current estimates the population of channels ranging from 80 to 250 in a typical patch. All tested substances shift the position of activation curves to the right. The examples of curve shifts for platensimycin and three analogs are shown in Figure 7A.

The pressure ramps were applied every two to five minutes and the p_0.5_ shifts were plotted as a function of time. The time courses of p_0.5_ for four tested substances are shown in Figure 7B. With QD-11, each sequential addition (arrows) leads to a rapid right shift with a gradual return toward the initial position. This relaxation of the initial shift suggests equilibration of the lateral pressure profile, implying permeation of the intercalated substance to the other side of the membrane. For QD-11, the process of initial unilateral insertion into the membrane appears to be five to six times faster than the process of flipping across the membrane. QD-6 and QL-3 show similar time courses of insertion, but less pronounced relaxation after each addition. PL and its analog QD-11 show not only faster insertion at the highest bulk concentrations (0.03 and 0.1 mM) but also faster relaxation. This suggests that massive intercalation in one leaflet generates tension in the opposite leaflet, which likely creates a lateral pressure gradient across the membrane that drives permeation. The time courses of p_0.5_ obtained in similar experiments with G258, G262, and G271 compounds are shown in Supplementary Figure S4.

**Figure 8:**
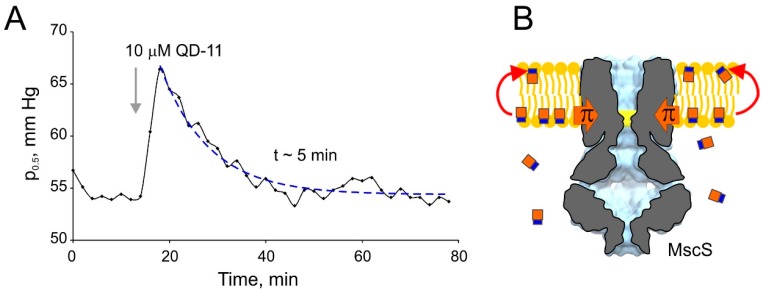
The kinetics of MscS midpoint relaxation in response to a single injection of QD-11 (**A**); The return of the activation curve back to its initial position with characteristic time of about 5 min is interpreted as a result of QD-11 redistribution between the membrane leaflets, as shown in (**B**), which likely leads to equalization of pressure profile. The time course signifies the kinetic of drug permeation across the membrane.

Having hypothesized that slow midpoint relaxation is associated with drug redistribution to the outer leaflet, we monitored the complete process of membrane relaxation to a single injection. The time course of midpoint relaxation after a single injection of QD-11 is shown in Figure 8A. The process was found to resemble an exponential relaxation, fit with a single exponent and found to have a characteristic time of 5.1 min (ranging between 4 and 8 min in four different trials). Figure 8B depicts the possible process of redistribution that partially equalizes the drug concentrations and lateral pressure in the two leaflets of the membrane, thus relieving the asymmetric pressure on the gate of MscS. We presume that the relaxation process reflects the slow establishment of a steady-state diffusional gradient of the drug across the patch.

The dose-response curves were constructed from cumulative up-shifts of p_0.5_ observed with each sequential addition of the drug and ignoring the decline phase after each injection. The p_0.5_ dependences on drug concentration for all six substances are shown in Figure 4C. The intercepts of fitting lines produce log*K*_mem_ between 5.0 and 6.1. The patch-clamp data (log*K*_mem_) is consistent with the monolayer data (log*K*_lip_) for the analogs, with the exception of PL and QD-11, for which the log*K*_lip_ values were higher (Table 2). One possible explanation is that the bulky non-planar side chains impart a larger cross-sectional area, which may relatively easily intercalate into the expanded monolayer. However, when the substance inserts into a full bilayer membrane, it induces additional stretching of the opposite leaflet, which opposes insertion. The two analogs with heterocyclic side chains, G258 and G262, exhibited moderate log*K*_mem_ of approximately 5.6, whereas for G271 this parameter was 6.1; the higher affinity for the membrane could be attributed to the extensive ring structures on the sidechains of G271 imparting larger surfaces for VdW interactions.

The bottom row in Table 1 presents minimal inhibitory concentrations (MIC) for all of the substances assayed with gram-positive and gram-negative bacteria [19]. Remarkably, platensimycin which is the most biologically active compound, resides in the middle of the hydrophobicity range and shows moderate *K*_mem_ compared to its analogs. The second most active compound is QD-11, which shows the highest surface activity, propensity to phospholipids, and affinity for the native bacterial membrane. Despite the fact that the heterocyclic G271 partitioned into the membrane very well, and can be docked well into FabF, it had virtually no antimicrobial activity.

## 3. Discussion

This study of membrane partitioning of platensimycin and its six synthetic analogs was motivated in part by computational docking to their intracellular target (FabF), which predicted that affinities of QD-11, G258, and G271 may be similar to that of platensimycin. Yet, among the six analogs only QD-11 was active against bacteria. We hypothesized that the inactivity of G258 and G271 could be caused by low membrane permeability, whereas the low activity of QL-3, QD-6, and G262 could be due to the combined effect of low affinity for FabF and possibly low membrane permeability. Indeed, inactivity of some engineered drugs even with perfectly designed pharmacophores may be due to either low membrane permeability (if too hydrophilic), or sequestration in lipids (if too hydrophobic). For this reason, the development of methods that could trace substances of interest directly in the membranes of their target organisms is of importance. These methods may help tune the balance between hydrophobic and hydrophilic properties of a new drug. By comparing the partitioning coefficients of these seven drugs to the air/water interface, monolayers formed from *E. coli* lipids, and native *E. coli* membranes, we observed the same trends among the analogs, but somewhat different values. The differences in values can be attributed to the differences in membrane and membrane-mimic systems.

Mechanosensitive channel MscS, naturally embedded in the cytoplasmic membrane of *E. coli* (with homologs in most known bacteria), readily detects partitioning of amphipathic drugs in the cytoplasmic leaflet of the membrane [14]. The mild gain-of-function mutant A98S used in this work is a more sensitive sensor of lateral pressure asymmetry than WT MscS. It activates at lower tension and exhibits a larger fractional shift of p_0.5_ upon asymmetric intercalation of a well-characterized test substance such as butyl paraben (Figure 1). By no means can this application of the patch-clamp technique be considered a “high-throughput” approach; patches are less stable in the presence of amphipaths, and a skilled patch-clamper could perhaps collect a complete dataset for one or two drugs (Figure 7) on a good day. Yet the technique can play its role in determining why a promising drug that displays a high affinity to its target in the test tube is inactive during *in vivo* experiments.

As illustrated in a cartoon in Figure 1, the channel detects the difference in pressures/tensions between the leaflets, in addition to the absolute value of lateral pressure. The channel gate is approximately at the level of phosphates of the inner phospholipid leaflet of the membrane [32] and the additional lateral pressure created by the substance intercalating in that leaflet requires extra tension to activate the channel. As the concentration of intercalating substance is increased, p_0.5_ progressively increases. The intercept of the p_0.5_/p_0.5 – control_ curves with the concentration axis reasonably approximates 1/*K*_mem_, but we cannot interpret the slope of the curve the same way as we can interpret the slope of Gibbs isotherms as molecular area [5]. This can be considered a drawback precluding quantitative thermodynamic analysis. However, it is this “differentiating” property that permits the detection of the redistribution kinetics of the intercalating substance between the two leaflets, *i.e.*, the process of permeation. The “flipping” across the bilayer results in an equilibration of pressure profiles on both sides of the membrane and this is read by the system as the midpoint relaxation back to its initial value. The kinetics of permeation appears to be slower than the process of initial unilateral incorporation. The exact time course of permeation is characteristic of each individual substance. Importantly, shorter characteristic time of permeation across the membrane for platensimycin and QD-11 (Figure 7 and Figure 8) directly correlate with the high antimicrobial activity. Redistribution of G258 seemed to also be relatively fast, but activation midpoints recorded with G262 or G271 did not return back within the observation time (Supplementary Figure S4); these heterocyclic substances had no biological effect.

As illustrated in Table 2, computational predictions of hydrophobicity generally underestimate membrane propensity for most of the analogs. Ranking by the percent polar surface area emphasizes the higher hydrophobicity of QD-11, but does not make a large distinction between some of the other substances (Table 2, Column 2). The segmental properties of polar and apolar regions are taken into account in the algorithms used in *ALOGPS 2.1* and *Molinspiration* software (Columns 3 and 4). However, these programs do not handle substances with dissociable groups such as carboxyls or amines, thus the calculations are presented for the uncharged forms of all compounds. LogD computed with *Chemicalize* provides oil-water partitioning coefficient with the account of ionizable groups, but these values are consistently two-three orders of magnitude lower than computed for uncharged forms, but are conspicuously five orders of magnitude lower than experimentally observed. This suggests that when entering the membrane these substances may change their protonation state, thus increasing their lipid affinity.

The data summarized in Figure 4 and Table 2 shows that all three experimental techniques give comparable partitioning coefficients. The differences point to the different components of the total thermodynamic force driving the substances out of the aqueous phase and into the membrane. These are hydrophobicity, amphipathicity (propensity toward the interface dictated by separation of polar and apolar groups), and favorable interactions with the phospholipids.

Estimations of log*K*_AW_ based on tensiometry measurements underestimate compound partitioning into the membrane (except for QD-11) because they take into account primarily the expulsion of the substance from the aqueous phase (the hydrophobic effect). Monolayer partition coefficients (*K*_lip_) are consistently higher by one to two orders of magnitude than air-water partition coefficients (*K*_AW_), indicating that favorable interactions with lipids contribute to the affinity, contrasting the monolayer environment from the bare air-water interface (see discussion of this subject in Seelig [31] and Suomolainen [5,30]). This difference is especially significant for the two less hydrophobic heterocyclic compounds G258 and G262; their log*K*_AW_ (air-water) is 4.2 *vs.* log*K*_lip_ (monolayer-water) of 5.9. It appears that their planar heterocyclic groups with large solvent-accessible area make VdW interactions with lipids a decisive contribution. The carboxyls on dihydroxyl benzoic acid in all compounds are predicted to be in a dissociated form in water (pKa~4) and this would make the molecules much more hydrophilic. Yet, the hydrophobicity estimations (log*K*_OW_) based on uncharged forms (Table 2, Columns 3 and 4) provide closer values to log*K*_AW_, log*K*_lip_, and log*K*_mem_ than LogD. This suggests that the dissociated carboxyl in the interface- or lipid-adsorbed configuration somehow remains exposed to water. Alternatively the carboxyl, after acquiring a proton, is intermittently “dragged” into the membrane with the rest of the molecule by apolar interactions, stabilizing the protonated form. We attempted to measure drug intercalation at acidic pH, however the patches were unstable below pH 6.0.

If we consider the experimental log*K*_mem_ measured via patch-clamp as a parameter reflecting all physical factors driving drugs into the membrane (hydrophobicity, favorable interactions with lipids, and the necessity to overcome lateral pressure of lipids [30,33]), we can say that lipid monolayers are an adequate system as a membrane mimic to study drug partitioning since the log*K*_lip_ it provides is almost identical to the log*K*_mem_ for most of the substances. Measured affinities in the monolayer (half-bilayer) system depend on lipid packing areas and corresponding lateral pressures (compare Columns 7 and 8 in Table 2). The small discrepancy between the monolayer and patch-clamp data is obvious for PL and QD-11, the two analogs characterized with the largest non-planar sidechains [34]. The discrepancy between the monolayer and the patch-clamp data may be due to the following reasons: (1) the larger cross-sectional area of these compounds make it more difficult to overcome lateral pressure of the entire bilayer and intercalate into the membrane in patch-clamp experiments, making the effective log*K*_mem_ lower than log*K*_lip_; (2) reaching true equilibrium in a compressed monolayer experiment with these compounds may require longer times; (3) without the second membrane monolayer in the Langmuir system the z-positions of adsorbed drugs (especially platensimycin and myrtenal compounds) may be different; if the drug resides close to the lipid-air interface, it would not perturb the monolayer as much as the bilayer since the lateral pressure distributions in the two systems are different [28]; (4) an important factor might be coupling between the two leaflets in the complete bilayer, in which substance intercalation in one leaflet should also produce work of expansion of the opposite leaflet since the two are coupled through the common midplane. Effectively, lateral compressibility of the single monolayer might be higher, which would harbor grafted amphipathic molecules more easily.

## 4. Experimental Section

### 4.1. Synthesis of Platensimycin Analogs

Platensimycin analogs were synthesized following published procedures [19,35]. Briefly, compound **1** was reacted with excess aldehyde and sodium cyanoborohydride to form ester 2. The ester group in compound 2 was hydrolyzed into the carboxylic acid 3, using sodium hydroxide, followed by hydrochloride acid. The synthesis and primary characterization of QL-3, QD-6, and QD-11 has been described previously [19]. The structural NMR data for the previously unpublished compounds G258, G262, and G271 are shown in the Supplementary data (Figures S5–S13).

### 4.2. Computations of Polarity and Partial Charges

Optimized structures, electrostatic potential maps, and partial charges were obtained using Gaussian 09 [36] at B3LYP/6-31G(d) level. The solvent effect (water) was taken into account using a polarizable continuum model (PCM). EPIWEB 4.1, ALOGPS 2.1 and *molinspiration* software were used to calculate the octanol-water partition coefficient (*K*_OW_) and polar surface area.

### 4.3. Tensiometry and Langmuir Monolayer Experiments

Surface tensions of subphase solutions with varying concentrations of platensimycin analogs were quantified using the Wilhelmy method with a piece of filter paper (Whatman, No. 1, 10.5 mm wide and 0.25 mm thick) used as a probe. The subphase buffer consisted of 200 mM KCl, 10 mM MgCl_2_, 5 mM CaCl_2_, 5 mM HEPES (Sigma-Aldrich, St. Louis, MO, USA) titrated to a pH of 7.4 with KOH. The pressure sensor (model 601, NIMA, Coventry, UK) was calibrated using a 100 mg weight. Water surface tension was measured to be −72 mN/m, after calibration. The sensor was hence zeroed, with further surface tension readings giving positive values for surface pressures. The surface activity and values for molecular area at the surface of the air-water interface (As), the partition coefficient at the air-water interface of the surface active molecule (*K*_AW_), and the membrane partitioning coefficient (*K*_mem_), were determined as described previously [5,15].

All Langmuir monolayer experiments were done with a two-barrier rectangular 22 cm × 6 cm trough (MicroTrough XS, Kibron Inc., Helsinki, Finland) placed in an Airclean hood. A Dyneprobe metal alloy needle was used as the Wilhelmy plate. Lipids used were *E. coli* total polar lipid extract from Avanti Polar Lipids (Alabaster, AL, USA) dissolved in chloroform or hexane to a final concentration of 0.2 mg/mL. Distribution of lipids onto the subphase was accomplished using a gastight 50 µL Hamilton^®^ syringe (Hamilton, Reno, NV, USA). The subphase buffer was identical to the tensiometry buffer. Pressure—Area isotherms (monolayers) were performed at room temperature (~20 °C) from 114 to 18 cm^2^ at a barrier rate of 20 mm/min. For each analog, isotherms were measured at least four times at every concentration.

### 4.4. Strains, Spheroplast Preparation, and Electrophysiology

A pB10b vector was used to house and express the *mscS* gene in the triple-knockout *E. coli* strain MJF465 (*mscS^−^, mscL^−^, mscK^−^*) [37]. The A98S MscS mutant, generated and partially characterized previously [21], was chosen for its higher sensitivity to inner leaflet drug partitioning.

Spheroplasts were generated as described previously [38,39,40]. The experimental bath solution contained 400 mM sucrose, 200 mM KCl, 50 mM MgCl_2_, 5 mM CaCl_2_, and 5 mM HEPES and was titrated to pH 7.4 with KOH. Membrane patches were obtained on borosilicate glass pipettes and recorded at 30 mV pipette voltage set by a microelectrode amplifier. The midpoint of activation for channels in each patch was determined at two-minute intervals for the duration of the experiment via a 1 s ramp of saturating pressure. Analogs of platensimycin were pre-diluted in either ethanol or bath solution and perfused into the bath to achieve the desired concentration; control injections of equivalent volumes of ethanol had no effect on the midpoint of activation. Negative pressure (suction) was applied with the HSPC-1 high speed pressure clamp apparatus from ALA Scientific. Programming of the pressure protocols and data analysis was performed with the PClamp10 suite (Axon Instr., Foster City, CA, USA). The principle of lateral pressure measurements using two experimental systems, lipid monolayers and patch-clamp, is illustrated in Figure 1.

### 4.5. Interfacial Partitioning Data Analysis

The analysis of interfacial adsorption data was performed in the general framework of the Gibbs equation

(1) $$d\pi =\sum {\text{Γ}}_{i}d\mu_{i}$$

where dπ represents a small change in surface pressure, Γ_i_ is surface excess of component i and dμ_i_ is the small change of chemical potential of that component in the aqueous phase. For one adsorbing component, in an ideal case the Gibbs isotherm takes the form

(2) dπ=RTΓd ln C

where surface excess is defined as the surface density of molecules Γ = 1/(*N_A_a_S_*), N_A_ is Avogadro’s number and *a_S_* is the area requirement for the molecule at the surface. The adsorbing substance gradually saturates the surface; the process that can be approximated by a Langmuir isotherm:

(3) $$\text{Γ}={\text{Γ}}_{\infty }\ln\frac{K_{\mathrm{AW}}\cdot C}{1+K_{\mathrm{AW}}\cdot C}$$

Thus, beyond a certain bulk concentration, Γ approaches a constant value of Γ_∞_ and in that range of concentrations a linear relationship holds:

(4) $${\text{Γ}}_{\infty }=(1/\mathrm{RT})\mathrm{d\pi}/\mathrm{d ln C}$$

To evaluate the partition coefficient for an air-water interface (*K*_AW_) and monolayers (*K*_lip_), the integral form of this equation attributed to Szyszkowski and previously applied to monolayers by Seelig and coworkers [41] was used:

(5) $$\text{π}=\mathrm{RT}{\text{Γ}}_{\infty }\ln(K_{\mathrm{AW}}\text{C}+1)$$

As was shown in Suomolainen 2004 [5], the x-intercept of the fitting line of experimental π-lnC curve would approximate the inverse partitioning coefficient 1/*K*_AW_, measured at the air-water interface.

In Langmuir experiments, the value of the bulk-monolayer (*K*_lip_) partitioning coefficient was determined in a similar way, using the deviations of surface pressure of monolayers measured at constant area. Initially, we attempted determining the Δπ(lnC) dependence starting from the suggested monolayer-bilayer equivalence pressure of 35 mN/m achieved at 68 Å^2^/molecule (for *E. coli* lipids) [14]. However, at this compression, strongly intercalating substances produced pressure shifts beyond the collapse pressure of 45–47 mN/m, which forced us to score Δπ from surface pressure of 20 mN/m achieved at ~80 Å^2^/molecule. *K*_lip1_ obtained in this expanded state was than corrected for the lateral pressure increase from π_1_ = 20 to π_2_ = 35 mN/m, which has the same form as Equation (7) in [5]:

(6) $$\ln(K_{\mathrm{lip}2})=\ln(K_{\mathrm{lip}1})-({\text{π}}_{2}-{\text{π}}_{1})a_{\text{d}}N_{A}/RT$$

Here *a*_d_ is the area taken by the drug in the plane of the monolayer.

Similarly, the bulk-membrane partitioning coefficient (*K*_mem_) was estimated from the values of relative increase in MscS activation pressure midpoint (p_0.5_) measured in patch-clamp experiments at different concentrations of intercalating agents by linear fit of Δp_0.5_ as a function of log(*C*_d_).

To estimate *a*_d_ we assumed equilibrium between the drug in the subphase and in the monolayer and equated its chemical potentials in the two phases:

(7) $$\mu_{0}+RT\ln(\chi_{d})=\mu_{0}^{\sigma }+RT\ln(\chi_{d}^{\sigma })+\pi \cdot a_{d}N_{A}$$

The right part with indexes σ represents the surface parameters of standard chemical potential ${\text{µ}}_{0}^{\text{σ}}$ and drug mole fraction ${\text{χ}}_{\text{d}}^{\text{σ}}$, whereas the left part represents the bulk parameters. Defining ln*K*_lip_ = ${\text{µ}}_{0}^{\text{σ}}$ −µ_0_ one can rearrange this equation as

(8) $$\ln K_{lip}-\ln\chi_{d}^{\sigma }-\ln\chi_{d}=\frac{\pi \cdot a_{d}N_{A}}{RT}$$

Differentiating with respect to π gives

(9) $$-RTd(\ln\chi_{d}^{\sigma })/N_{A}d\pi =a_{d}$$

Thus the molecular area of the drug defines the slope of ${\text{χ}}_{\text{d}}^{\text{σ}}$, with which the drug re-partitions back to the subphase as surface pressure π increases. We further assume that at a given surface pressure, insertion of *n*_d_ drug molecules into the film comprised of *n*_L_ lipid molecules with initial area *A*_0_, should dilate the film by Δ*A*.

(10) $$\frac{n_{d}}{n_{L}}=\frac{\Delta A}{A_{0}}\cdot \frac{a_{L}}{a_{d}}$$

The parameter *a*_L_, the area per lipid at that particular pressure, is taken from the control pressure-area isotherm with no drug. The mole fraction of the drug in the monolayer ${\text{χ}}_{\text{d}}^{\text{σ}}$ = *n*_d_/(*n*_d_ + *n*_L_) is related to the extent of monolayer dilation Δ*A*

(11) $$\Delta A=a_{d}\cdot n_{d}=\frac{a_{d}n_{L}\cdot \chi_{d}^{\sigma }}{1-\chi_{d}^{\sigma }}$$

On the assumption that ${\text{χ}}_{\text{d}}^{\text{σ}}$ is small, Δ*A* is proportional to the mole fraction of the drug. In Equation (4) we substitute and obtain

(12) $$-kTd(\ln\Delta A)/d\pi =a_{d}$$

Thus, the molecular area of the drug can be determined from the logarithmic slope of Δ*A* on surface pressure. Once we know *a_d_*, we can introduce a correction to *K*_lip_ due to lateral pressure change (Equation (2)). In addition, we can estimate the molar ratio of drug-to-lipid, the mole fraction ${\text{χ}}_{\text{d}}^{\text{σ}}$ and the partitioning constant. From the extent of monolayer dilation at a constant surface pressure (chosen to be 20 mN/m), the apparent bulk-monolayer partitioning coefficient *K*_lip_ could be independently estimated as $K_{lip}=\frac{\chi_{d}^{\sigma }}{\chi_{d}}$, where ${\text{χ}}_{\text{d}}^{\text{σ}}$ = *C*_d_/55 represents the drug mole fraction in the subphase.

## 5. Conclusions

The development of a patch-clamp based technique to estimate the partitioning of extrinsic amphipathic substances into the cytoplasmic membrane of *Escherichia coli* using mechanosensitive channel MscS as a sensor of lateral pressure opens new possibilities for characterizing membranotropic properties of natural or newly synthesized compounds. It is definitely not a high-throughput system, yet here we provided it as simple proof of principle. The comparison of partitioning coefficients for a particular set of substances obtained in different systems emphasizes not only the differences of the systems, but different components of thermodynamic forces driving the substances into and across the membranes. For platensimycin and the six synthetic analogs with different sidechains, surprisingly, we found very comparable membrane partitioning coefficients, suggesting that not only hydrophobicity and amphipathicity, but also favorable VdW interactions with the lipids, drive membrane partitioning and permeation.

## Supplementary Materials

Supplementary materials can be found at http://www.mdpi.com/1422-0067/16/08/17909/s1.
