# Supplementary material for: Membrane Affinity of Platensimycin and Its Dialkylamine Analogs
Source: Int J Mol Sci. 2015 Aug 4;16(8):17909–32. doi: 10.3390/ijms160817909 (PMC4581228; doi:10.3390/ijms160817909)
Supplement: Supplementary file 1 [file ijms-16-17909-s001.pdf]

Supplementary Information

PL

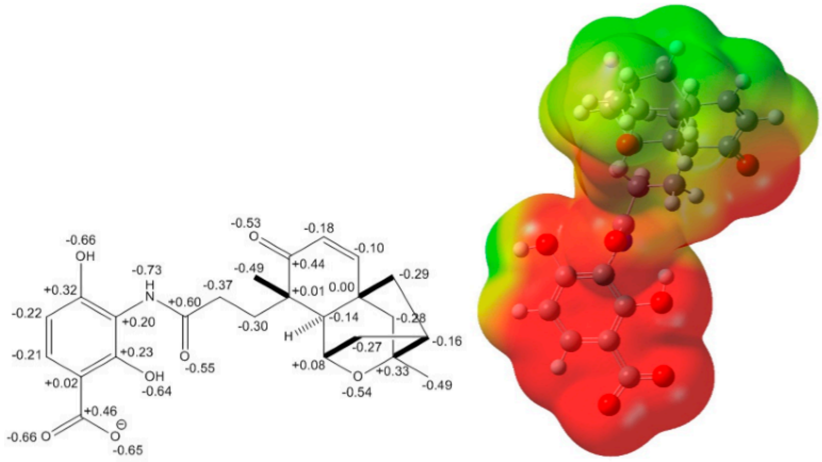

QL-03

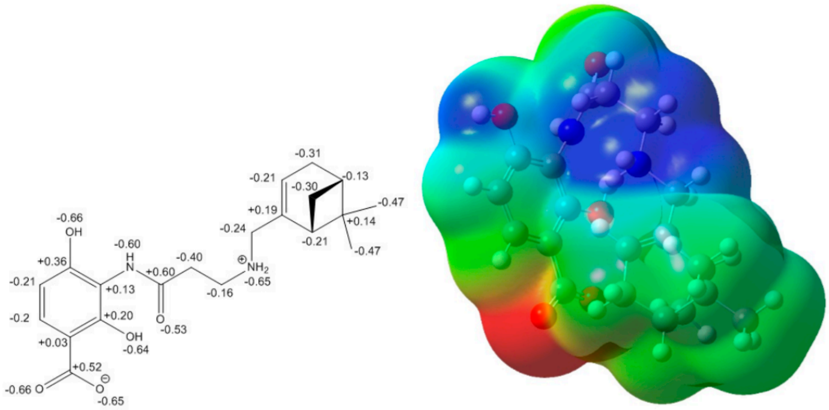

QD-11

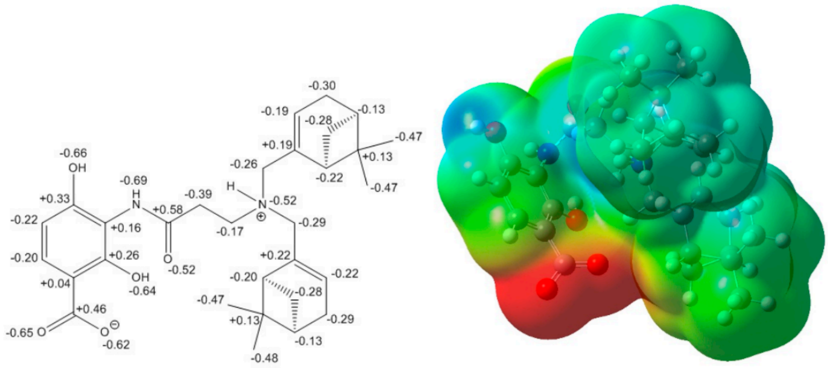

QD-06

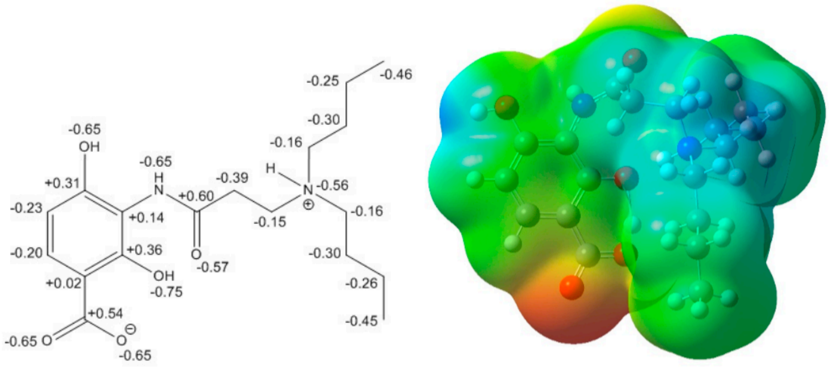

(A)

Figure S1. *Cont.*

G258

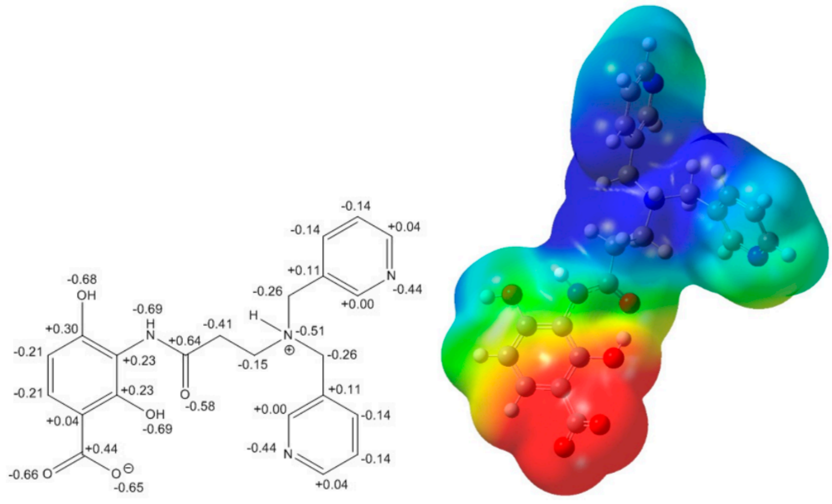

G262

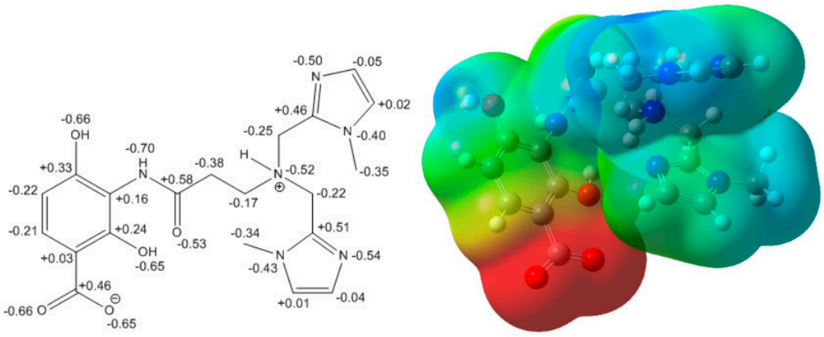

G271

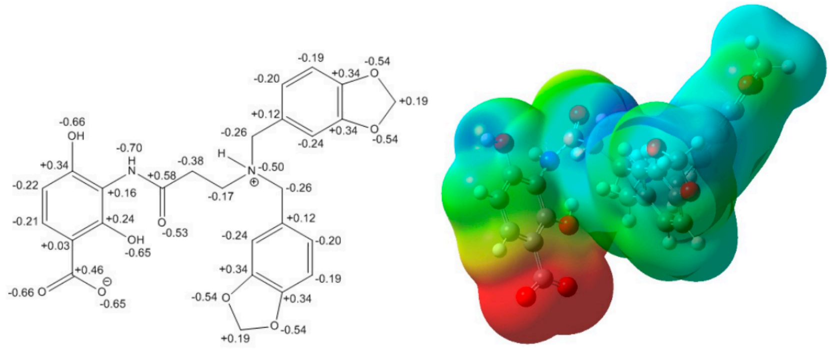

(B)

Figure S1. (**A**) Structures and partial charge distributions for ionized Platensimycin and three of its zwitterion analogs computed in implicit water; (**B**) Structures and partial charge distributions for three zwitterion heterocyclic Platensimycin analogs computed in implicit water. The red and blue colors represent negatively and positively charged regions of the molecule, respectively; green represents neutral.

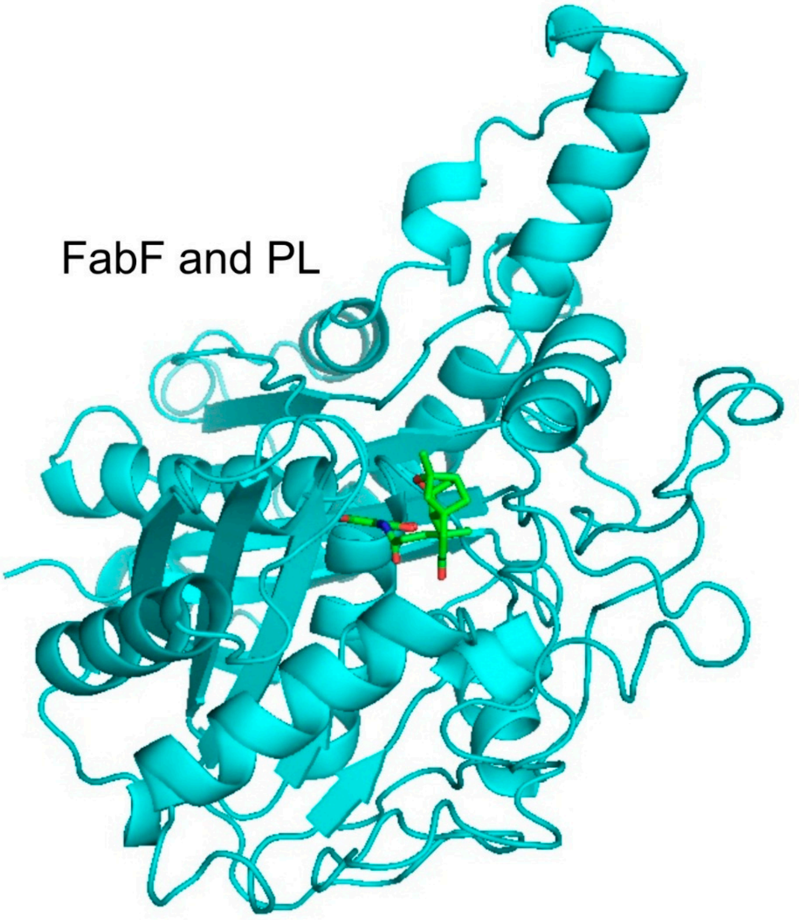

(**A**)

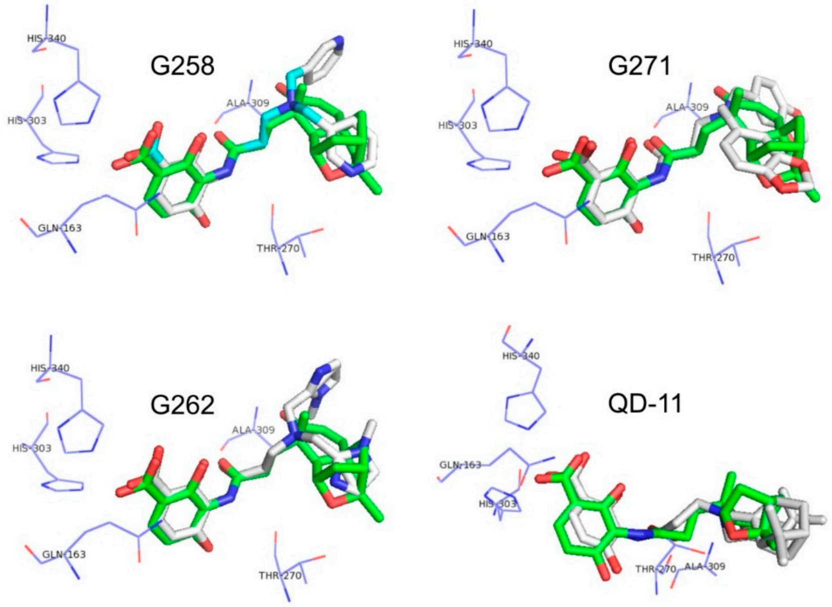

(**B**)

Figure S2. (**A**) The docked conformation of platensimycin (green) within FabF binding pocket as represented by the crystal structure of FabF (C163Q) (PDB ID: 2GFX) (blue); (**B**) The docked conformations of G258, G262, G271 and QD-11 within the binding pocket of FabF (C163Q) (PDB ID: 2GFX). The analogs are all shown in white allowing comparison with the docked conformation of platensimycin (green).

Docking Calculations

Docking calculations were performed using Autodock Vina 1.1.1 [22]. A grid box large enough to encompass the ligand in the binding pocket was chosen. The exhaustiveness value was set as 16 in the Autodock calculations and the rest of the parameters were used at their default values. The ligand PDB files were prepared with ChemDraw. Autodock Tools 1.5.4 was used to convert the PDB files into PDBPT files for the Autodock vina calculations. The top-ranked conformations were selected for analysis. Results were visualized using PyMOL viewer version 1.3 (Schrodinger, L.L.C. Unpublished work).

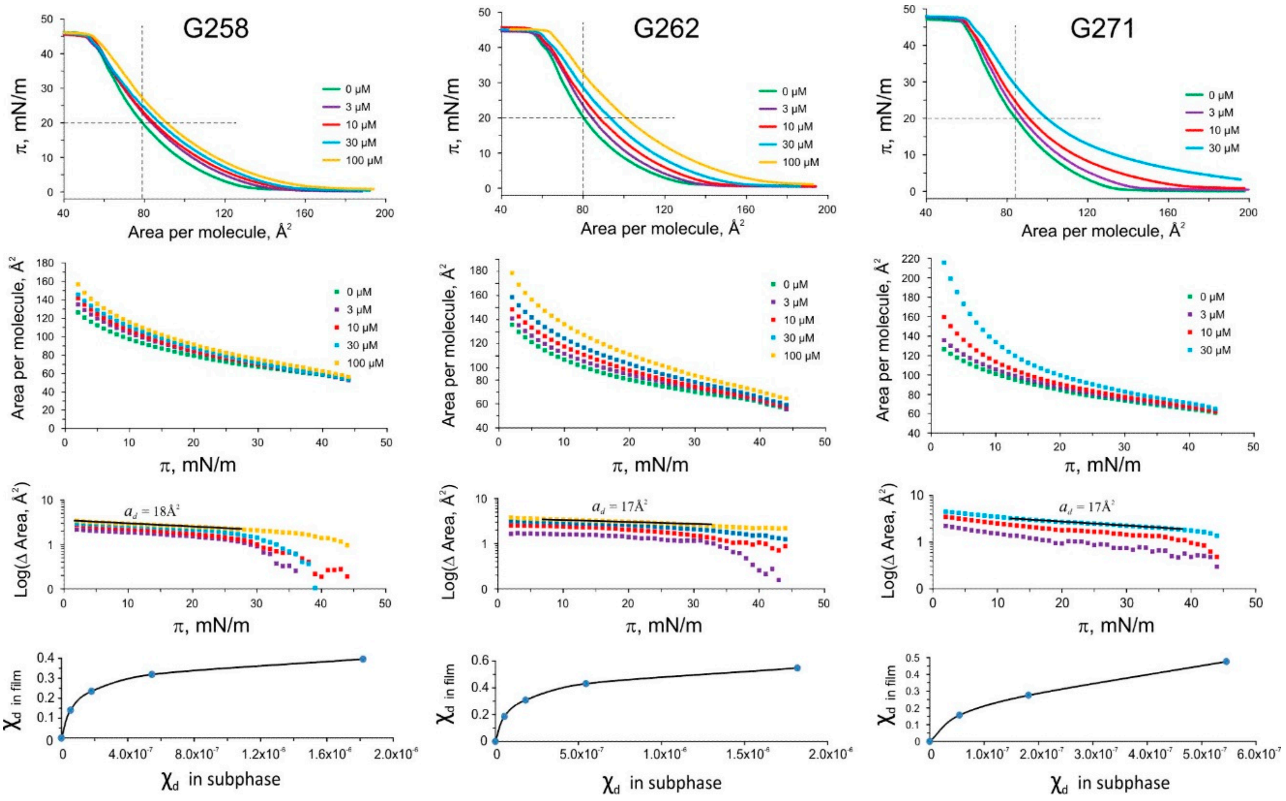

Figure S3. Pressure-area (π-A) isotherms for three heterocyclic analogs of platensimycin. Green curves or symbols in all cases are control isotherms taken without intercalating agents. The upward-right shifts of π-A curves with concentration (**upper row**) indicate intercalation of the amphipathic substance from the subphase into the monolayer. Plots of the same curves in inverted A-π coordinates (**second row**) and differences from control presented in semi-log scale (**third row**) with the linear fits and estimated molecular areas for the drugs. Mole fraction of drugs in the monolayers plotted *versus* mole fraction in the subphase (**bottom**).

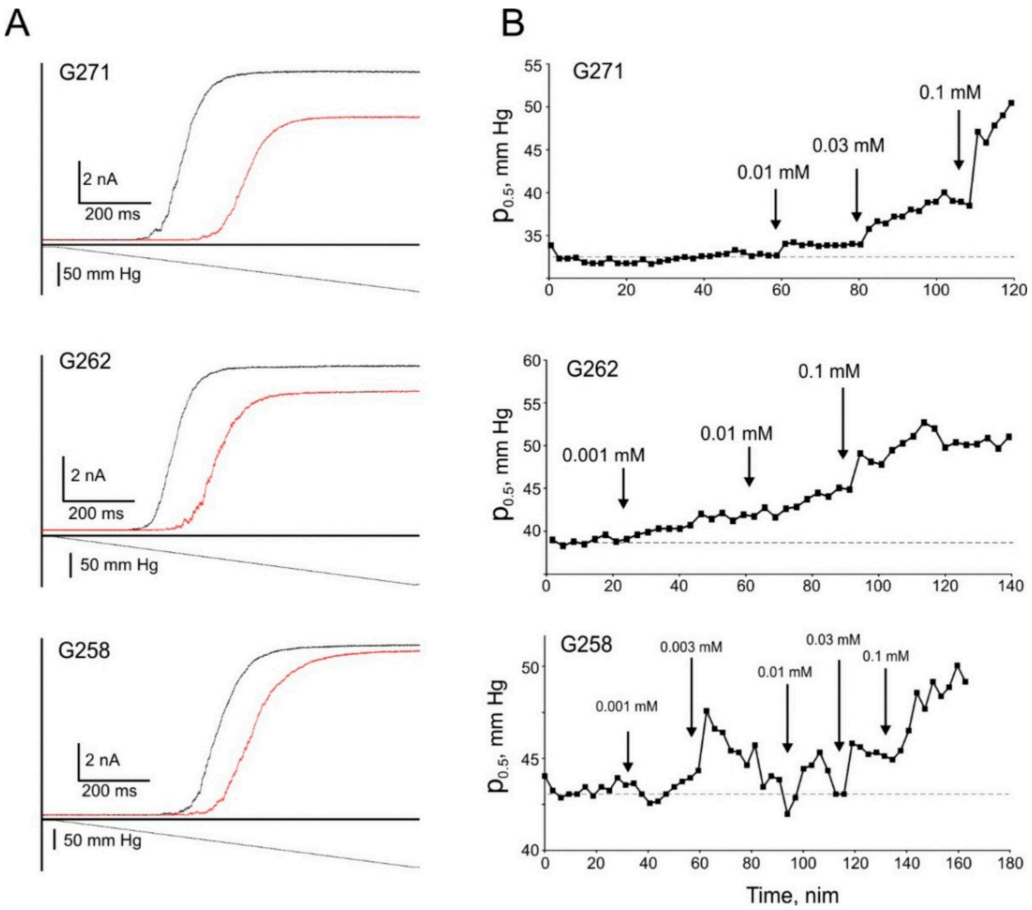

Figure S4. Ramp responses of A98S MscS following additions of the three hetrocyclic platensimycin analogs and the time dependencies of the resulting midpoint shift ($p_{0.5}$). The shift of the activation curve of A98S MscS toward higher tension after exposure to the denoted compounds is shown (**A**); The activation midpoints of the curves ($p_{0.5}$) were then plotted against time (**B**). Arrows indicate the point in time at which the given concentration of the compound was added to the bath. Cumulative amplitudes of $p_{0.5}$ shifts (shown by grey arrows in the plot for QD-11, Figure 7, main text) were used to plot and fit concentration dependencies in Figure 4E,F (main text).

Chemical Synthesis and Characterization of New Compounds

All reagents were obtained commercially unless otherwise noted. Reactions were performed using oven-dried glassware under an atmosphere of argon. Air- and moisture-sensitive liquids and solutions were transferred via syringe or stainless steel cannula. Dry tetrahydrofuran (THF) and diethyl ether (Et_2O) were distilled over sodium prior to use and dry dichloromethane (DCM) was distilled over CaH_2 prior to use. Thin-layer chromatography (TLC) was performed on Merck Kieselgel 60 F254 plates. Visualization of the developed chromatogram was accomplished by UV light or by staining with $KMnO_4$ solution. Chromatographic purification of products was accomplished using flash column chromatography on silica gel (230 × 400 mesh). Compounds purified by chromatography on silica gel were typically applied to the absorbent bed using the indicated solvents conditions with a minimum amount of added dichloromethane as needed for solubility. Solvents were removed from the reaction mixture or combined organic extracts by concentration under reduced pressure using an evaporator

with bath at 30–40 °C. Elevated temperatures were obtained using thermostat-controlled silicone oil baths. Low temperatures were obtained by ice bath or by mixing dry-ice with organic solvents. NMR spectra were measured on Bruker AV-400, Bruker DRX-400 (^{1}H at 400 MHz, ^{13}C at 100 MHz), Bruker DRX-500 (^{1}H at 500 MHz, ^{13}C at 125 MHz) or Bruker AVIII-600 (^{1}H at 600 MHz, ^{13}C at 150 MHz). Data for ^{1}H-NMR spectra are reported as follows: chemical shift (ppm, relative to residual solvent peaks or indicated external standards; s = singlet, d = doublet, t = triplet, q = quartet, dd = doublet of doublets, td = triplet of doublets, m = multiplet), coupling constant (Hz), and integration. Data for ^{13}C NMR are reported in terms of chemical shift (ppm) relative to residual solvent peak. Mass spectra (MS) and high resolution mass spectra (HRMS) were recorded by JEOL AccuTOF-CS (ESI positive, needle voltage 1800–2400 eV).

G258

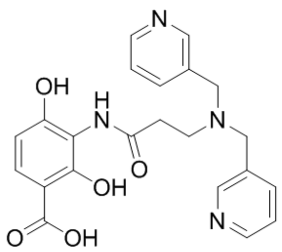

Figure S5. Chemical structure of G258.

^{1}H NMR (500 MHz, d_4-MeOD) δ ppm 8.55 (s, 2H), 8.40 (s, 2H), 7.98 (d, *J* = 7.8 Hz, 2H), 7.67 (d, *J* = 8.7 Hz, 1H), 7.44–7.31 (m, 2H), 6.41 (d, *J* = 8.7 Hz, 1H), 5.52 (s, 1H), 3.77 (s, 4H), 2.96 (t, *J* = 6.2 Hz, 2H), 2.76 (t, *J* = 6.1 Hz, 2H). ^{13}C NMR (125 MHz, d_4-MeOD) δ ppm 174.1, 173.1, 157.0, 155.4, 148.9, 147.1, 138.1, 135.4, 128.8, 123.8, 112.4, 109.3, 107.2, 54.9, 49.9, 33.8. HRMS (ESI+, *m*/*z*) calculated for $C_{22}H_{23}N_4O_5$ $[M + H]^+$ 423.1668, found 423.1682.

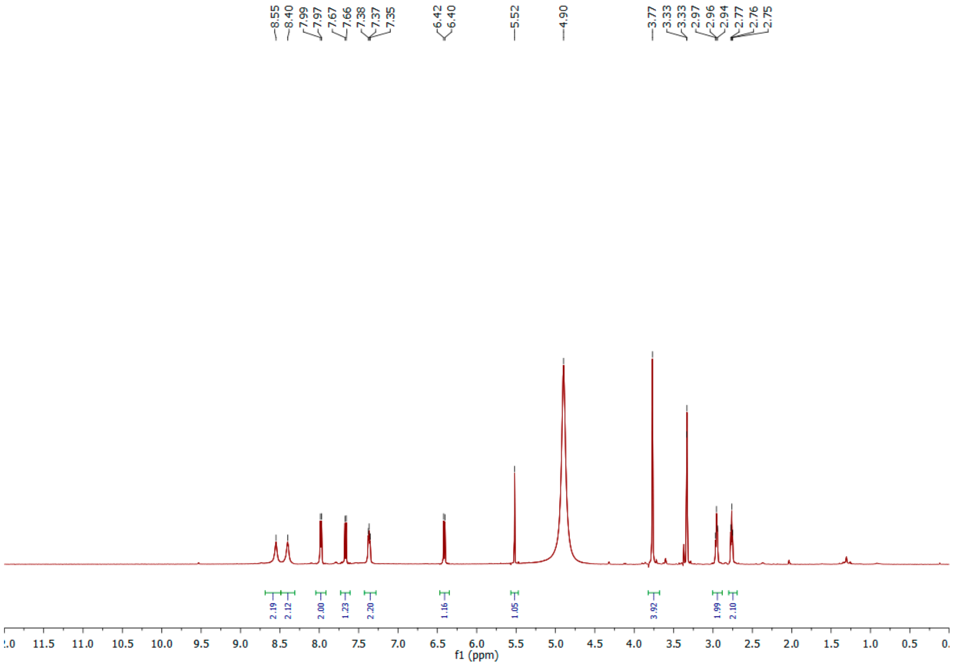

Figure S6. ^{1}H NMR spectrum of G258.

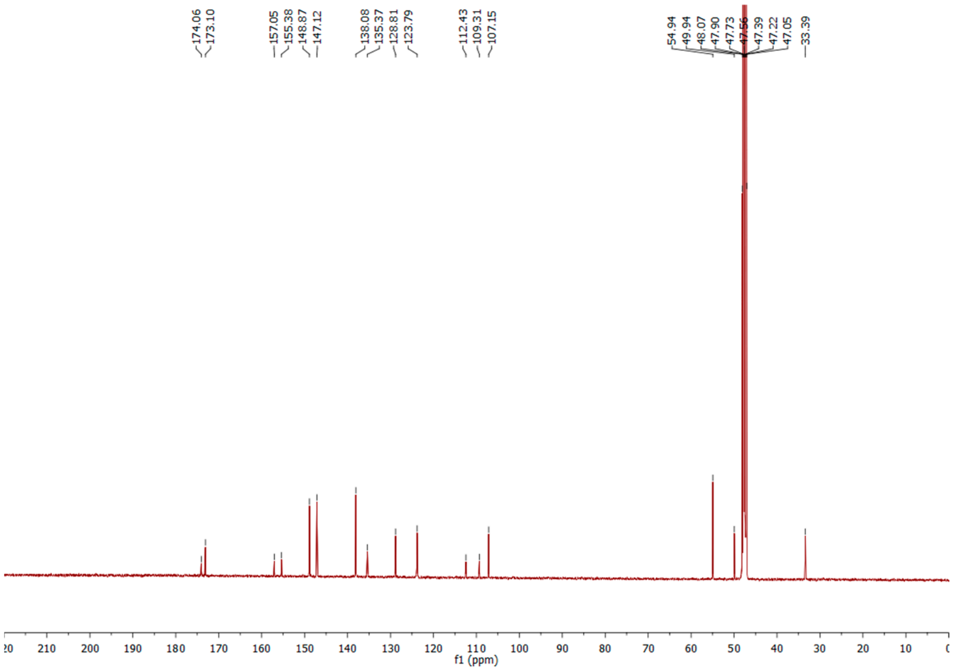

Figure S7. ^{13}C NMR spectrum of G258.

G262

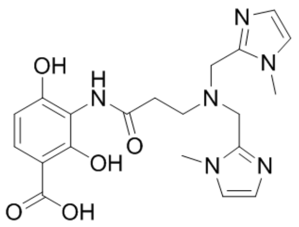

Figure S8. Chemical structure of G262.

^{1}H NMR (400 MHz, *d*4-MeOD) δ ppm 7.67 (d, *J* = 8.7 Hz, 1H), 7.27 (s, 2H), 7.17 (s, 2H), 6.39 (d, *J* = 8.7 Hz, 1H), 4.07 (s, 4H), 3.75 (s, 6H), 3.08 (t, *J* = 5.1 Hz, 2H), 2.77 (t, *J* = 5.1 Hz, 2H). ^{13}C NMR (100 MHz, *d*4-MeOD) δ ppm 174.6, 173.4, 158.9, 156.9, 144.8, 130.0, 123.8, 121.4, 112.3, 110.2, 106.7, 51.7, 49.2, 33.4. HRMS (ESI+, *m*/*z*) calculated for $C_{20}H_{25}N_6O_5$ [M + H]$^+$ 429.1886, found 429.1876.

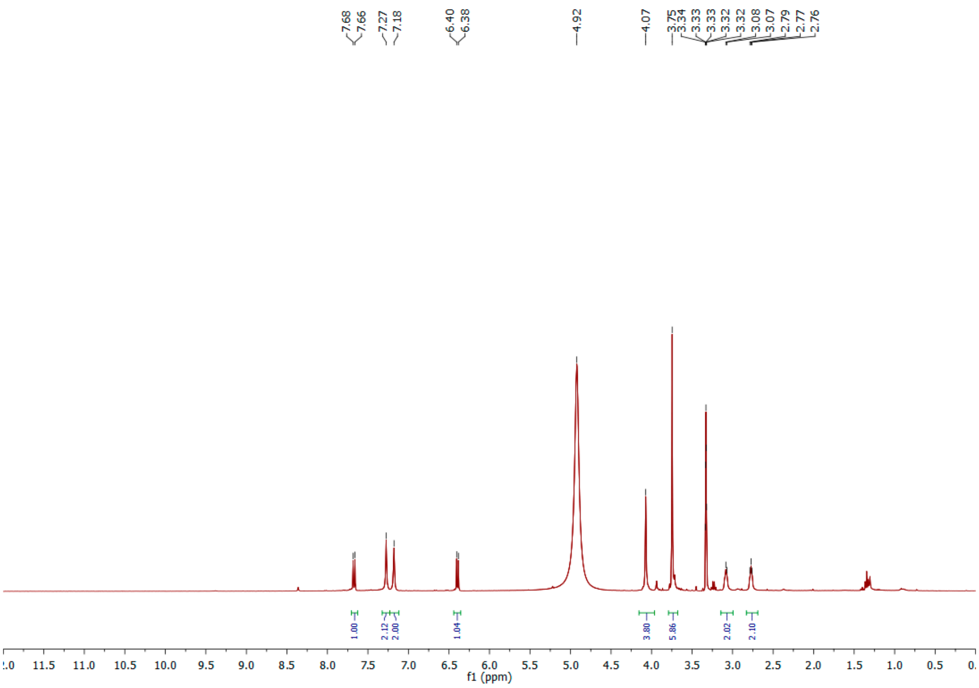

Figure S9. ^{1}H NMR spectrum of G262.

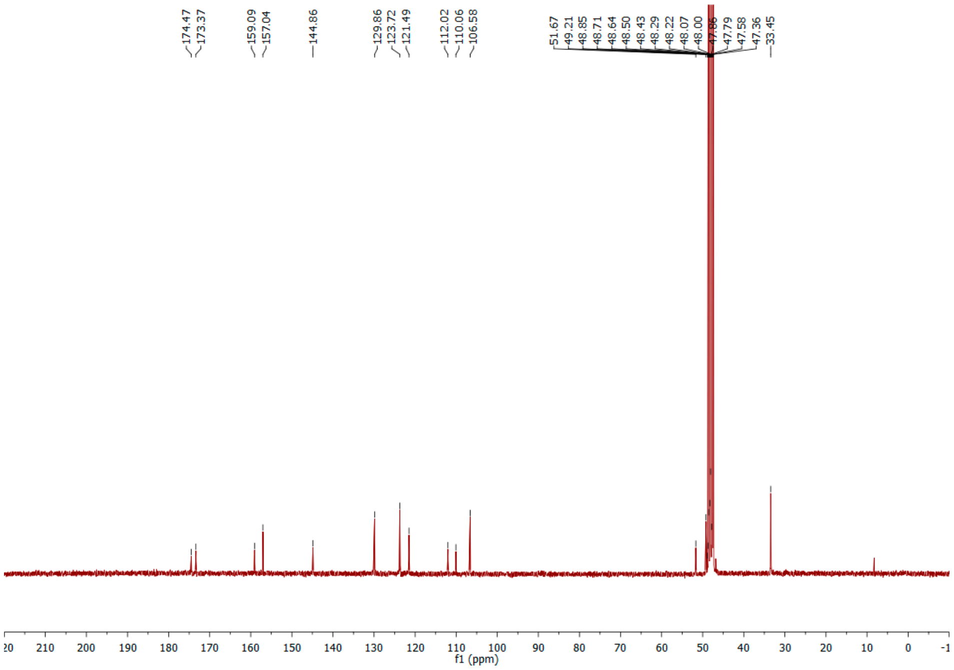

Figure S10. ^{13}C NMR spectrum of G262.

G271

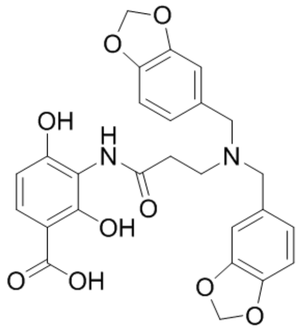

Figure S11. Chemical structure of G271.

^{1}H NMR (400 MHz, d_4-MeOD) δ ppm 7.66 (d, J = 8.7 Hz, 1H), 6.93 (s, 2H), 6.86 (d, J = 7.9 Hz, 2H), 6.73 (d, J = 7.9 Hz, 2H), 6.35 (d, J = 8.7 Hz, 1H), 5.89, (s, 4H), 3.71 (s, 4H), 2.99 (t, J = 6.3 Hz, 2H), 2.70 (t, J = 6.2 Hz, 2H). ^{13}C NMR (100 MHz, d_4-MeOD) δ ppm 179.0, 173.3, 157.4, 155.3, 148.4, 147.7, 130.6, 129.2, 123.2, 112.6, 109.9, 107.9, 107.2, 101.4, 57.8, 49.5, 32.5. HRMS (ESI+, m/z) calculated for $C_{26}H_{25}N_2O_9$ $[M + H]^+$ 509.1560, found 509.1553.

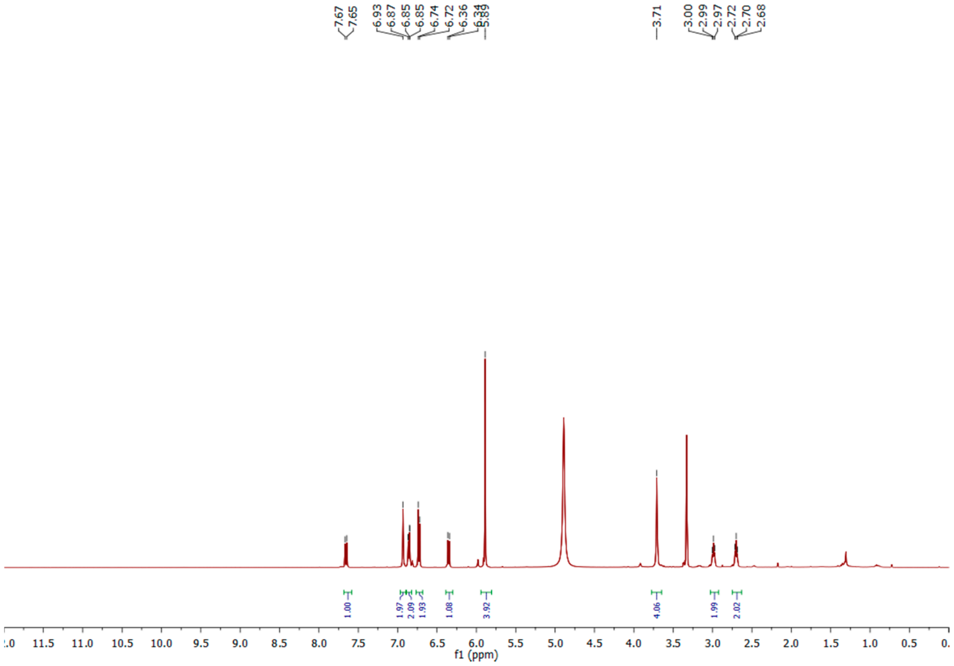

Figure S12. ^{1}H NMR of G271.

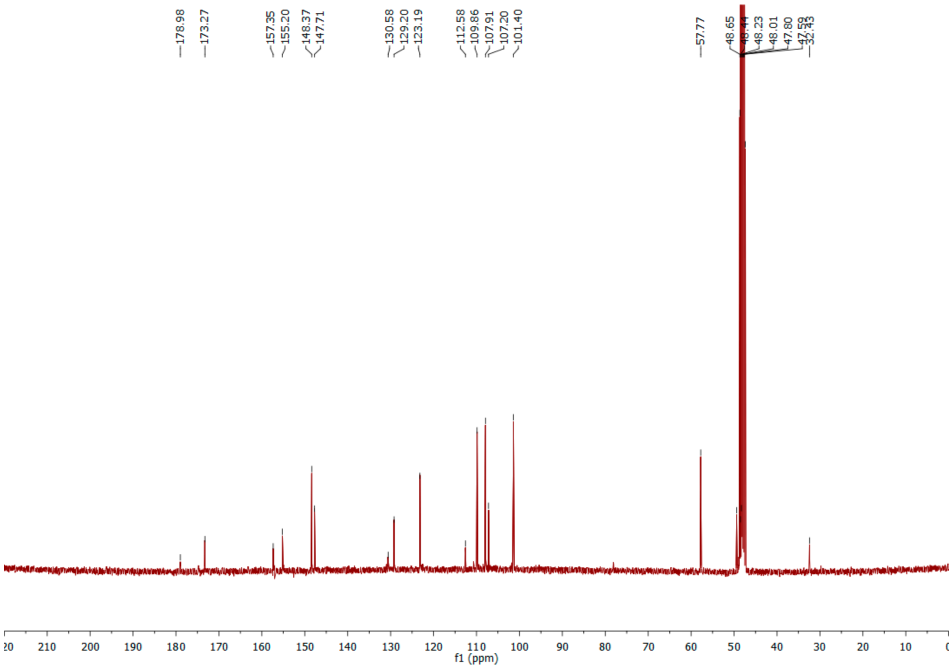

Figure S13. ^{13}C NMR of G271.
